# Inhibition of Anaplerosis Attenuated Vascular Proliferation in Pulmonary Arterial Hypertension

**DOI:** 10.3390/jcm9020443

**Published:** 2020-02-06

**Authors:** Mathews Valuparampil Varghese, Joel James, Cody A Eccles, Maki Niihori, Olga Rafikova, Ruslan Rafikov

**Affiliations:** Department of Medicine, Division of Endocrinology, University of Arizona College of Medicine, Tucson, AZ 85721, USA; mathewsv@email.arizona.edu (M.V.V.); joeljames@deptofmed.arizona.edu (J.J.); codyeccles@email.arizona.edu (C.A.E.); mniihori@deptofmed.arizona.edu (M.N.)

**Keywords:** anaplerosis, pyruvate carboxylase inhibitor, oxaloacetate, pulmonary arterial hypertension, pyruvate dehydrogenase, glycolysis, glucose oxidation

## Abstract

Vascular remodeling is considered a key event in the pathogenesis of pulmonary arterial hypertension (PAH). However, mechanisms of gaining the proliferative phenotype by pulmonary vascular cells are still unresolved. Due to well-established pyruvate dehydrogenase (PDH) deficiency in PAH pathogenesis, we hypothesized that the activation of another branch of pyruvate metabolism, anaplerosis, via pyruvate carboxylase (PC) could be a key contributor to the metabolic reprogramming of the vasculature. In sugen/hypoxic PAH rats, vascular proliferation was found to be accompanied by increased activation of Akt signaling, which upregulated membrane Glut4 translocation and caused upregulation of hexokinase and pyruvate kinase-2, and an overall increase in the glycolytic flux. Decreased PDH activity and upregulation of PC shuttled more pyruvate to oxaloacetate. This results in the anaplerotic reprogramming of lung vascular cells and their subsequent proliferation. Treatment of sugen/hypoxia rats with the PC inhibitor, phenylacetic acid 20 mg/kg, starting after one week from disease induction, significantly attenuated right ventricular systolic pressure, Fulton index, and pulmonary vascular cell proliferation. PC inhibition reduced the glycolytic shift by attenuating Akt-signaling, glycolysis, and restored mitochondrial pyruvate oxidation. Our findings suggest that targeting PC mediated anaplerosis is a potential therapeutic intervention for the resolution of vascular remodeling in PAH.

## 1. Introduction

Pulmonary arterial hypertension (PAH) is considered as a fatal vascular disease progressing to right ventricular (RV) dysfunction and failure if untreated. In PAH, vascular remodeling and proliferative changes narrow the lumen of pulmonary arteries, causing increased vascular resistance [1]. Approved therapies specifically function as vasodilators to reduce the symptoms but fail to attenuate remodeling and the disease [2]. The inadequacy of existing PAH therapies is connected to the composite nature of pathological events found at the stage of the developed disease. Pulmonary vascular cell dysfunction is considered as an initiator on the pathogenesis of PAH [3]. Yet, vascular cell proliferation in PAH remains incompletely elucidated. In PAH pathogenesis, understanding the molecular mechanisms that initiate vascular cell transition to a highly proliferative phenotype, could help for earlier diagnostics and improve the treatment of PAH.

Previously, it was reported that smooth muscle cells (SMCs) isolated from the pulmonary vessels of PAH rats showed enhanced glycolysis [3] under aerobic environments (Warburg effect), considered as a major contributor of metabolic reprogramming. However, increased glycolysis alone is inadequate to encounter the complete metabolic requirement of highly proliferating cells. It is now known that proliferative cells must go through different metabolic reprogramming to encounter their higher energy, and building blocks need for proliferation in PAH [4]. The tricarboxylic acid (TCA) cycle offers a significant source of substrates for amino acids, carbohydrates, and lipids biosynthesis [5]. In proliferative cells, the sustained activity of the TCA cycle needs the supplementation of carbon intermediates. Anaplerosis helps to replenish carbon intermediates by carboxylation of pyruvate to oxaloacetate through pyruvate carboxylase (PC) and by glutaminolysis (glutamine deamidation by glutaminase). The carboxylation reaction of PC is achieved by its ATP dependent binding to biotin which results in transferring a carboxyl group to pyruvate, thus converting it to oxaloacetate (OAA) [4,6]. PC mediated OAA production is considered as the initial phase of the gluconeogenesis pathway [7]. Previous studies reported a marked increase in the anaplerotic and gluconeogenic pathways in correlation with elevated PC expression in vascular proliferative cells [8]. Therefore, we hypothesize that inhibition of PC could prevent anaplerotic reprogramming and vascular proliferation in pulmonary vascular cells and this could attenuate PAH pathogenesis. The selective inhibition of PC activity in vascular cells may provide a better target for treating pulmonary vascular cell remodeling in PAH.

## 2. Experimental Section

### 2.1. Human Subjects

De-identified human plasma samples consisted of patients with a diagnosis of diabetes mellitus (DM) group (*N* = 12), left heart disease (HD) group (*N* = 11), and idiopathic pulmonary arterial hypertension (IPAH) group (*N* = 11) were obtained through the Center for Disparities in Diabetes, Obesity and Metabolism and Health Sciences Biorepository at the University of Arizona, and from Pulmonary Hypertension Breakthrough Initiative (PHBI). PHBI rigorously phenotyped PAH patients. Patient’s age was (mean ± standard deviation): 41.1 ± 15.7 (IPAH group), 41.8 ± 13.7 (DM group), and 50.2 ± 10.9 (HD group). All PAH patients were receiving therapy. No patients from the IPAH cohort had DM; one IPAH patient had valvular LV heart disease. The University of Arizona and PHBI study protocols were approved by the Institutional Review Boards of the participating sites in the network (IRB00001995), and all sites were adherent to the requirements of the U.S. Federal Policy for the Protection of Human Subjects (45 CFR, Part 46), and supported the general ethical principles of the Declaration of Helsinki.

### 2.2. Rat Model of PH

Female Sprague Dawley rats (200–250 g) were purchased from Charles River (Wilmington, MA, USA) and used for this study. Animals were kept at 22 °C, 12-h light:dark cycle, and had free access to water ad libitum and standard rodent food. All experimental protocols were approved by the University of Arizona Institutional Animal Care and Use Committee. Generally, PAH was induced by subcutaneous injection of SU5416 (50 mg/kg), followed by 3 weeks of hypoxia (10% O_2_) and 2 weeks of normoxia. This study included four animal groups: Control—untreated group, SU1 —SU5416 treatment with 1 week hypoxia; SU5—SU5416 treatment with 3 week hypoxia followed by 2 week normoxia; iPC (SU5 + PC) inhibitor, phenylacetic acid (PAA) (P16621, Sigma-Aldrich, St. Louis, MO, USA) treatment 20 mg/kg/every other day by intraperitoneal (i.p) injection starting after 1 week from disease induction by SU5416. The dose of PAA was selected based on the cell culture data, where higher dose of PAA stimulated Akt phosphorylation in pulmonary vascular cells.

### 2.3. Hemodynamic Measurement

Rats were anesthetized with Inactin 100 mg/kg i.p. (T133, Sigma-Aldrich, St. Louis, MO, USA). Right ventricular systolic pressure (RVSP) was assessed by a pressure transducer catheter (SPR-513, Millar Instruments, Houston, TX, USA), which was inserted into the right jugular vein and advanced into the right ventricle (RV) to monitor as described previously [9,10]. Briefly, the pressure transducer catheter was attached to a Millar Transducer Control Unit TC-510 and PL3504 PowerLab 4/35 data acquisition system (AD Instruments, Colorado Springs, CO, USA) to screen RV pressure for 30-min. After these animals were connected to a tracheal catheter ventilator system (Harvard Rodent Ventilator-683; Harvard Apparatus, South Natick, MA, USA), then opened the thorax and through the RV the lungs were flushed with 0.9% sodium chloride. Heart and lungs were dissected from animals; the RV free wall was parted from the left ventricle (LV), and the septum (S). Fulton index (RV/LV + S ratio) as a parameter of RV hypertrophy was assessed. After this, the left lungs were fixed in formalin and embedded in paraffin for histological studies. Remaining portions of the lungs were stored at −80 °C for further studies.

### 2.4. Histopathological Analysis

For the morphometric assessment of pulmonary vessels, 5-μm tissue sections were dewaxed and stained with hematoxylin and eosin (H&E) by HistoWiz Inc. (Brooklyn, NY, USA) using standard operating procedures and fully automated workflow system. Ten transversely sectioned pulmonary arteries (PA) (diameter < 300 μm to 50 μm) from each animal were selected randomly from the whole-slide 40X digitized image Aperio AT2 scanner (Leica Biosystems, Buffalo Grove, IL, USA)). Immunohistochemistry was performed on 5 µm sections on a Bond Rx autostainer (Leica Biosystems, Buffalo Grove, IL, USA) with heat mediated antigen retrieval using standard protocols. The sections were deparaffinized and incubated with primary antibodies against Ki67 (1:800) (ab15580 Abcam, Cambridge, MA, USA); Dab Rabbit H1 (pH 6) for 20 min. The morphometric evaluation was performed using a blinded grouping fashion. The wall thickness of the PA was assessed using the software Fiji ImageJ (Version-1.52p National Institute of Health, Washington, USA; http://fiji.sc/Fiji; in the public domain) [11].

### 2.5. Western Blot Analysis

A total of 20–40 mg of lung tissue was lysed in permeabilization buffer mixed with Halt^TM^ Protease and Phosphatase Inhibitor Cocktail (78444, Thermo Fisher Scientific, Rockford, IL, USA) using a FisherBrand Homogenizer-850. Then centrifuged the homogenate at 10,000 g for 10 min, and the supernatant was collected [12]. Membrane and cytosolic fractions were isolated using the FractionPREP^TM^ cell fraction kit (Biovision, Milpitas, CA, USA) according to the manufacturer’s instruction. Total protein concentration was assessed using the BCA protein assay kit (Thermo Scientific, Rockford, IL, USA). Then incubated tissue lysates with 6x Laemmli sample buffer (Boston Bioproducts Inc., Ashland, MA, USA), at 95 °C for 5 min, loaded on the 4–20% SDS-PAGE Mini-PROTEAN TGX Stain-FreeTM gels (Bio-Rad Laboratories Inc., Hercules, CA, USA) and separated by electrophoresis. Using the Trans-Blot Turbo transferring system (Bio-Rad Laboratories Inc., Hercules, CA, USA), protein bands were transferred and then blocked with 5% bovine serum albumin in Tris-buffered saline. Membranes were probed using antibodies against the following antibodies: Glut 4 (1:1000, 07-1404) from Millipore, pyruvate carboxylase (1:1000, Ab229267) from Abcam, pyruvate dehydrogenase (1:1000, 45-6600) from Invitrogen, phospho Akt (1:1000, Ser 473) (1:1000, 4060S), Akt (1:1000, 9272S), hexokinase 1 (1:1000, C35C4), hexokinase 2 (1:1000, 2867), lactate dehydrogenase-A (1:1000, 2012S), phosphor-glycogen synthase kinase (GSK) 3 β (Ser9) (1:1000, 9336S), GSK3 β (1:1000, 9315S) and glucose 6-phosphate dehydrogenase (1:1000, 8866S) from Cell Signaling Technology and pyruvate carboxykinase (1:1000, 14892-1-AP) from ProteinTech. The reactive bands were visualized by a chemiluminescent ChemiDocTM MP Imaging System (Bio-Rad Laboratories Inc., Hercules, CA, USA) and analyzed using Image LabTM software (Version 6.0.1, Bio-Rad Company, Hercules, CA, USA). The protein loading was normalized per total sample protein using stain-free gels as previously described [13]. This normalization is equal to housekeeping genes normalization and rigorously evaluated by Bio-Rad Company (Hercules, CA, USA; http://www.bio-rad.com/en-us/applications-technologies/stain-free-imaging-technology?ID=NZ0G1815) and by our lab in comparison with beta-actin normalization.

### 2.6. Pyruvate Dehydrogenase and Oxaloacetate Assay

Pyruvate dehydrogenase (PDH) activity was measured with PDH Activity Assay Kit (MAK183, Sigma-Aldrich, Saint Louis, MO, USA) using the provided protocol and all values were normalized with respect to total protein concentrations. Oxaloacetate concentration was measured with an oxaloacetate assay kit (MAK070, Sigma-Aldrich, Saint Louis, MO, USA) according to the manufacturer’s instruction and oxaloacetate concentration was expressed in nmol/mg protein.

### 2.7. Statistical Analysis

Statistical analysis was performed using GraphPad Prism version 7.04 (GraphPad Software, San Diego, CA, USA). The mean value (± SEM) was calculated for all samples, and significance was determined by either the unpaired *t*-test or analysis of variance (ANOVA). One-way ANOVA followed by Bonferroni multiple comparison tests were performed to compare the selected pairs of columns. The significance is calculated using a 95% confidence interval. Grubbs’ test was used to identify outliers using GraphPad outlier calculator (Alpha = 0.5) at https://www.graphpad.com/quickcalcs/Grubbs1.cfm.

## 3. Results

### 3.1. PC Inhibitor Attenuated Ventricular Pressure and Histological Changes

**Figure 1:**
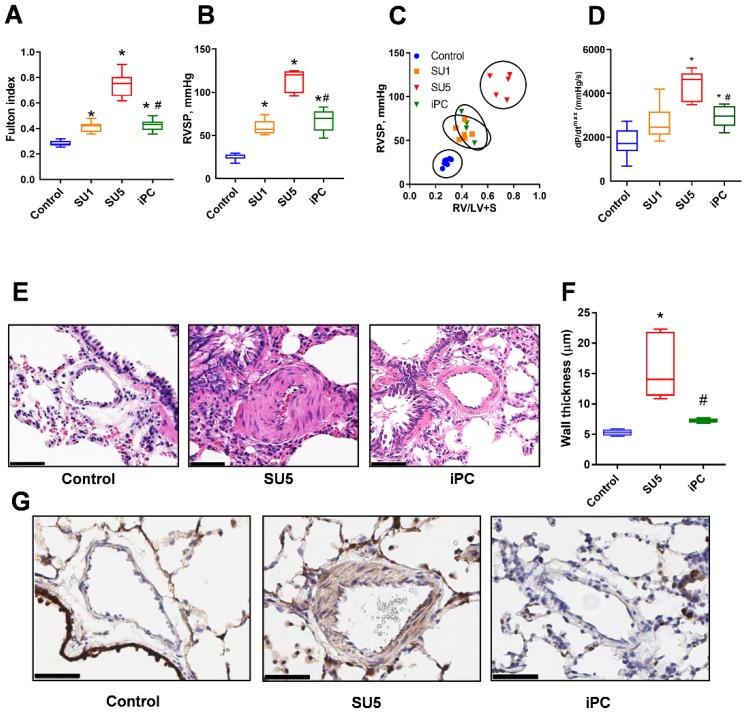
Pyruvate carboxylase (PC) inhibitor reduced hemodynamic and pathological changes in Sugen5416 treatment with three-week hypoxia followed by 2 week normoxia (SU5) rats and prevented pulmonary arterial hypertension (PAH) pathogenesis. (**A**) Right ventricular systolic pressure (RVSP) was significantly increased in SU5416 treatment with one-week hypoxia (SU1) and SU5 groups but PC inhibitor (iPC) treatment in SU5 groups controlled further increase in right ventricular (RV) pressure. (**B**) Fulton index, the ratio of RV to left ventricle and septum (RV/(LV+S)), was significantly increased in SU1 and SU5 groups and PC inhibitor treatment prevented its further increase from SU1 level. (**C**) Correlation analysis between RVSP and Fulton index showed a significant attenuation of the PAH phenotype in SU5 rats treated with the PC inhibitor. (**D**) SU5 groups showed increased dP/dtmax, but PC inhibitor significantly improved cardiac function and attenuated dP/dtmax. (**E**) hematoxylin and eosin (H&E) staining exhibited vascular fibrosis, constriction of pulmonary arteries (PA), and vascular proliferation in SU5 and these alterations were significantly attenuated with PC inhibitor treatment (scale–50 µm). (**F**) PA wall thickness was found to increase in the SU5 group, PC inhibitor treatment protected from the additional vascular remodeling. (**G**) Ki-67 immunohistochemical staining showed increased cellular proliferation in the media of the pulmonary artery in the SU5 group and PC inhibitor effectively prevented vascular remodeling and cellular proliferation (scale–50 µm). (**A**,**B**,**D**,**F**) expressed as box-and-whisker plot with median value ± min max, *N* = 5–6, * *p* < 0.05 versus control, # *p* < 0.05 versus SU5 by ANOVA.

In the SU5416/hypoxia PAH model, invasive hemodynamic measurements showed increased RV systolic pressure (RVSP) (Figure 1A) and as a result, the RV of PAH model demonstrated increased hypertrophic changes (Fulton index) (Figure 1B). Increased RVSP showed a significant correlation with the Fulton index (Figure 1C). dP/dt^max^ is a measure of myocardial contractility was increased SU5 PAH group, but the PC inhibitor treatment significantly improved cardiac function and prevented further increase in dP/dt^max^ (Figure 1D). The body weight was the same between control, sugen, and PC inhibitor groups (281 ± 10; 254 ± 7 and 271 ± 8 g, respectively). Inhibition of anaplerosis by PC inhibitor (PAA) treatment, which was administered one week after disease induction, significantly decreased the RV hypertrophy and attenuated the RVSP at Week Five, *p* < 0.001. These results demonstrate that PC inhibition can stop the further progression of PAH as indicated by RV pressure and Fulton index. Histological changes related to pulmonary artery remodeling were assessed by H&E staining [14]. Lung tissue of the PAH disease model showed concentric lesions of pulmonary arteries (PA). These histological alterations explain the cause of vascular obstruction and RV pressure overload in PA’s (Figure 1E,F). Inhibition of PC mediated anaplerosis prevented vascular proliferation in the lung tissue after sugen/hypoxic conditions. Immunohistochemical staining in lung tissue with Ki-67—a protein marker for cellular proliferation showed increased signal in SMCs of PA in the SU5 group. PC inhibitor treatment reduced vascular cell proliferation (Figure 1G). These observations suggest that anaplerosis inhibition can alleviate vascular remodeling and histopathological alterations and will ultimately help to regulate vascular flow back to normal. Previously, it was reported that PAA can induce inflammation [15]. Thus, we also checked the level of inflammatory markers in lungs in response to PC inhibition. Our data in Figure S1 indicate that E-Selectin, a marker of endothelial activation, was increased in the disease and PAA significantly attenuated it. Similarly, interleukin 1b (IL-1b) showed increase in sugen/hypoxia model and reduction with PC inhibitor. IL-6 and myeloperoxidase (MPO) did not respond neither to the sugen/hypoxia or treatment. Thus, the dose of PAA used in the study only resolved inflammation.

### 3.2. Inhibition of Akt Phosphorylation and Glucose Regulation by PC Inhibitor

**Figure 2:**
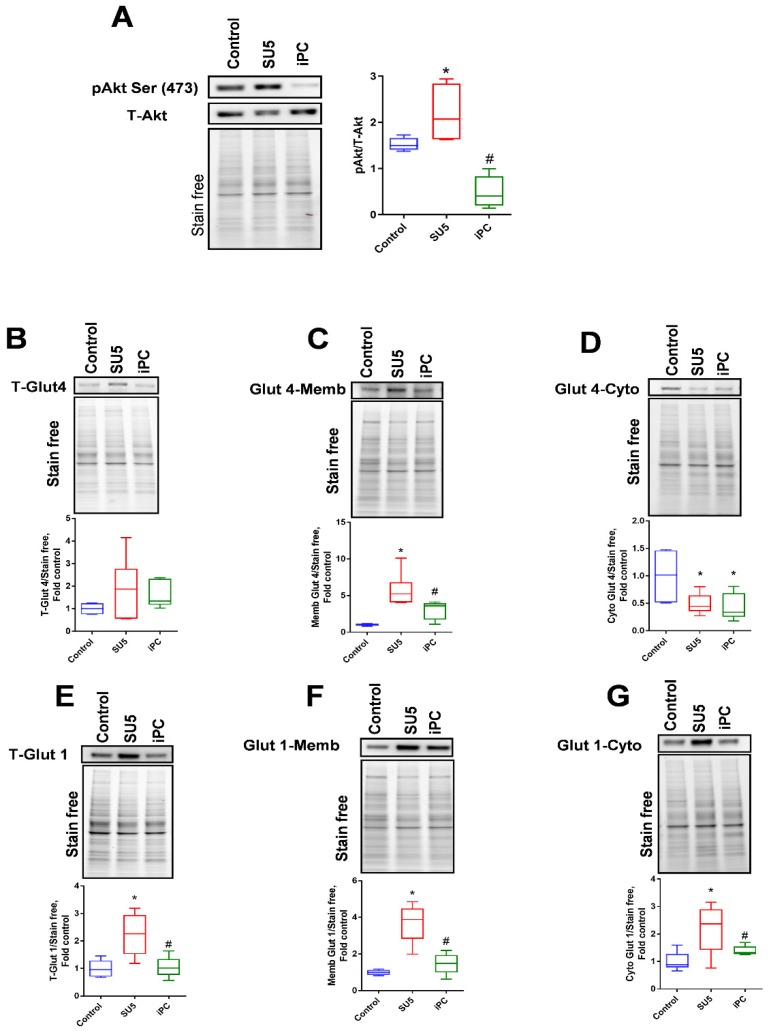
PC inhibitor reduced protein kinase B (Akt) phosphorylation and glucose transporters. (**A**) Akt phosphorylation ratio was significantly increased in the SU5 group was significantly downregulated with PC inhibitor treatment. (**B**) Glucose transporter Glut 4 (total) expression was not significantly altered, but (**C**) in the SU5 group, Glut 4 membrane expression was found increased and it was found decreased in (**D**) cytosolic fraction. PC inhibitor treatment significantly attenuated Glut 4 translocation from the membrane to the cytosol. Another glucose transporter, Glut 1, showed increased expression; (**E**) total glut 1 expression in whole lung lysate, (**F**) Glut1 expression in the membrane fraction and (**G**) Glut1 expression in the cytosolic fraction. Data expressed as box-and-whisker plot with median value ± min max, *N* = 5–6, * *p* < 0.05 versus control, # *p* < 0.05 versus SU5 by ANOVA.

Akt is a master regulator of glycolysis. Phosphorylation of Akt at Serine (S473) was reported as an important triggering factor in the pathogenesis of PAH [16]. In the present study, Akt activation in the sugen/hypoxic PAH model was found elevated with the upregulation of Akt phosphorylation at Serine-473 (Figure 2A). Interestingly, the inhibition of PC showed a drastic decrease in the phosphorylation of Akt. These results suggest that the inhibition of anaplerosis with PAA could regulate the Akt phosphorylation by downstream signaling cascade and controls further metabolic dysfunctions.

In our sugen/hypoxic PAH model, we screened the glycolytic metabolism which was highly increased in proliferative cells. Akt, an important activator of glucose transporters, can increase glucose uptake into the cells for glycolysis and oxidative phosphorylation. In our disease model, Glut4 membrane translocation was found to increase in correspondence with increased Akt phosphorylation (Figure 2B–D). Glut4 expression in the cytosol was found to decrease in the SU5 group. In conjunction with this, the other glucose transporter, Glut1, also showed increased expression in the disease model (Figure 2E–G). This could confirm the induction of glycolysis in PAH due to increased translocation of glucose transporters Glut4 and Glut1 on the membrane (Figure 2C,F). Interestingly, PC inhibitor treatment decreased Glut 4 and Glut 1 translocation back to the control level.

**Figure 3:**
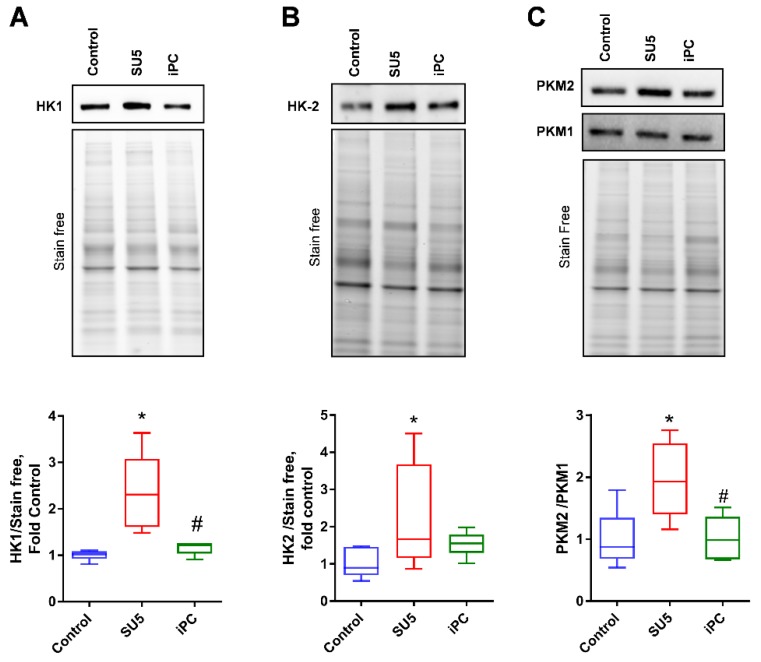
PC inhibitor attenuated increased glycolysis in PAH. Glycolytic enzymes (**A**) hexokinase-1 (HK1), (**B**) hexokinase-2 (HK2), and (**C**) pyruvate kinase 2 (PKM2), but not PKM1 showed increased expression in the SU5 PAH model. PC inhibitor treatment significantly attenuated glycolytic enzymes expression back to normal. Data expressed as box-and-whisker plot with median value ± min max, *N* = 5–6, * *p* < 0.05 versus control, # *p* < 0.05 versus SU5 by ANOVA.

Increased glucose concentration in the cytosol and activation of Akt signaling contributes to the upregulation of glycolysis. The initial rate-limiting enzymes of glycolysis (HK-1 and HK-2) were significantly increased in our PAH disease model (Figure 3A,B), indicating increased glycolysis. The final rate-limiting step of glycolysis is regulated by pyruvate kinase (PKM). Interestingly, our sugen/hypoxic disease model showed an increased PKM2/1 ratio in the lung tissue (Figure 3C). Increased expression of PKM-2 is associated with increased glycolytic metabolism and proliferative growth [17]. Importantly, the inhibition of PC decreased the altered glycolytic metabolism back to normal and thereby prevented the glycolytic shift in proliferating cells.

### 3.3. PC Inhibition on Glycogen Synthesis and Pentose Phosphate Pathway

**Figure 4:**
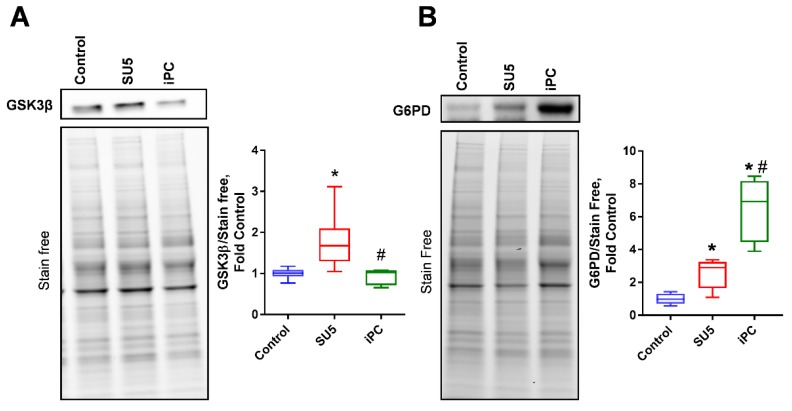
PC inhibitor balanced glycolysis by regulating Glucose 6-phosphate dehydrogenase (G6PD), and glycogen synthase kinase 3 beta (GSK3β). (**A**) GSK3β, an inhibitor of glycogen synthase, increased in the PAH model, was decreased with PC inhibitor treatment. (**B**) G6PD expression was significantly increased in SU5 and PC inhibitor treatment. Increased G6PD expression with PC inhibitor decreased glycolysis by increasing the pentose phosphate pathway. Data expressed as box-and-whisker plot with median value ± min max, *N* = 5–6, * *p* < 0.05 versus control, # *p* < 0.05 versus SU5 by ANOVA.

In the PAH model, glycogen synthesis was attenuated via the increased expression of glycogen synthase kinase-3β (GSK3β), which downregulates glycogen synthesis by inhibiting the glycogen synthase (Figure 4A). Inhibition of anaplerosis by blocking PC activity increased glycogen synthesis function to normal. Glucose 6-phosphate dehydrogenase (G6PD), the rate-limiting enzyme of the pentose phosphate pathway (PPP), was found to increase in the SU5 group (Figure 4B). PC inhibition also showed a rise in G6PD expression as a compensatory mechanism to control the increased glycolytic rate via shunting glucose-6-phosphate to PPP. These results suggest that PC inhibition attenuated the increased glycolytic metabolism in proliferating pulmonary vascular cells by upregulating parallel PPP and glycogenesis metabolism.

### 3.4. Inhibition of PC Inverted the Glycolytic Shift to Glucose Oxidation

**Figure 5:**
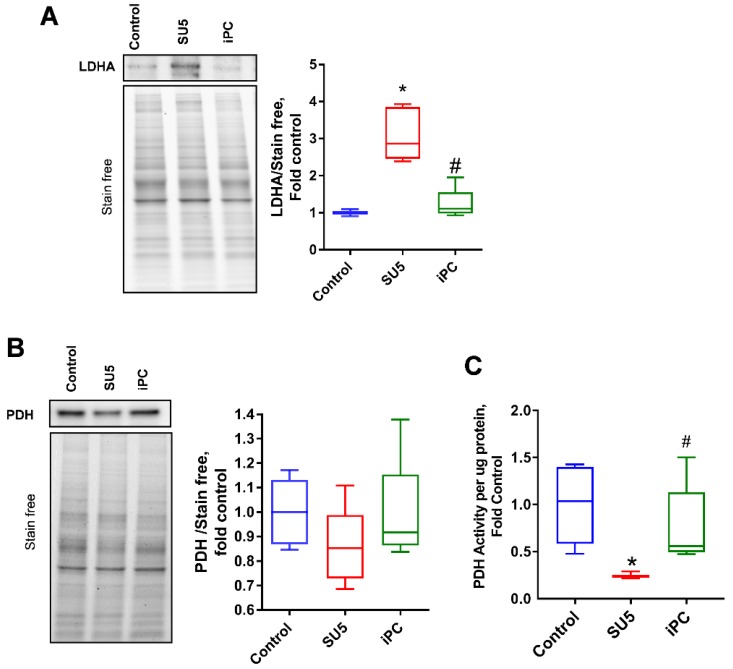
PC inhibitor controlled the balance between lactate dehydrogenase (LDH) and pyruvate dehydrogenase (PDH) in PAH. (**A**) Lactate dehydrogenase (LDHA) increased in the SU5 group was significantly reduced with PC inhibitor treatment. (**B**) Total PDH expression was not significantly altered, but (**C**) PDH activity significantly attenuated in the SU5 PAH group, and PC inhibitor treatment reduced this back to the control level. Data expressed as box-and-whisker plot with median value ± min max, * *p* < 0.05 versus control, # *p* < 0.05 versus SU5 by ANOVA.

The increased glycolytic rate in sugen/hypoxia results in the increased production of pyruvate. In the PAH disease model, increased pyruvate to lactate production was identified with the increased expression of lactate dehydrogenase (LDHA) (Figure 5A). This causes an increase in lactic acid production and, thereby, lactic acidosis, a favorable environment for cellular proliferation in PAH, and many cancers [18,19]. PDH is the enzyme that converts pyruvate to acetyl Co-A and supports oxidative phosphorylation. In the sugen/hypoxia disease model, PDH expression was not significantly altered, but its activity was found drastically decreased (Figure 5B,C). This indicates an impairment in the production of acetyl Co-A and decreased mitochondrial pyruvate oxidation. Interestingly, PC inhibition reduced LDHA expression and recovered the PDH activity back to the control level, which helps to reduce the glycolytic shift in pulmonary vascular cells.

### 3.5. Anaplerotic Reprogramming was Controlled with PC Inhibition

**Figure 6:**
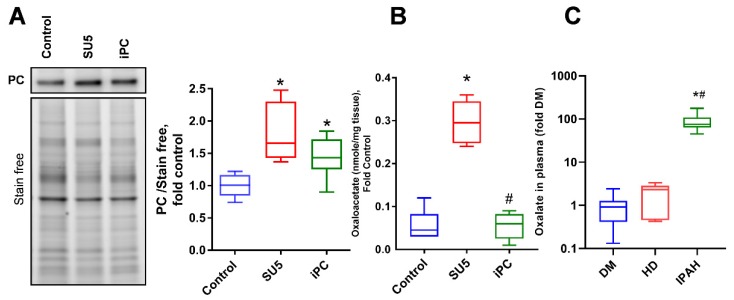
PC inhibitor controlled oxaloacetate (OAA) production in PAH. (**A**) Pyruvate carboxylase expression and its product. (**B**) Oxaloacetate was significantly increased in the PAH model. (**C**) Increased oxalate plasma concentration was identified in idiopathic PAH (IPAH) patients compared to diabetes mellitus (DM) or left heart disease (HD) patients. Data expressed as box-and-whisker plot with median value ± min max, *N* = 5–6, * *p* < 0.05 versus control or DM, # *p* < 0.05 versus SU5 or HD by ANOVA.

In proliferative cells, pyruvate metabolism was found to be associated with PC activation and the inhibition of PDH function [7]. Decreased PDH function is reported as one of the mitochondrial causes of PAH [20]. Inhibition of PDH activity decreased acetyl Co-A production from pyruvate, resulting in the upregulation of PC and increased OAA production from pyruvate in the TCA cycle; thus, upregulating the anaplerotic pathway (Figure 6A,B). PAH patients showed a 100 fold increase in oxalate concentrations, the product of OAA decomposition, in the plasma when compared to other disease conditions such as diabetes mellitus and left heart diseases (Figure 6C). In PAH conditions with pulmonary vascular remodeling, vascular cells may utilize OAA as a precursor for the production of biomolecules [21]. Thus, increased anaplerosis can cause pulmonary vascular cell proliferation in PAH. These results represent a metabolic shift from oxidative phosphorylation to anaplerosis and glycolysis during the disease, a favorable condition for cellular proliferation. PC inhibition decreased the oxaloacetate production, thus, preventing anaplerotic reprogramming. These results suggest that inhibition of PC can control the proliferative phenotype in pulmonary vascular cells by rebalancing the glycolytic shift to oxidative phosphorylation. 

## 4. Discussion

PAH is considered a vascular disease with increased pulmonary vascular remodeling via proliferation. Evaluation of the precise mechanisms responsible for the early switching of normal vascular cells to highly proliferative cells seems to be critical in developing effective treatment approaches. PAH animals showed increased pulmonary arterial SMC proliferation and vascular remodeling by anaplerotic reprogramming [3]. Specifically, PC activity contributes to anaplerosis and plays a major role in regulating cell growth and viability in proliferative cells that have transferred their metabolism from oxidative phosphorylation to glycolysis [22]. In the present study, we observed that inhibition of anaplerosis by PC prevented metabolic reprogramming in sugen/hypoxia PAH rats.

**Figure 7:**
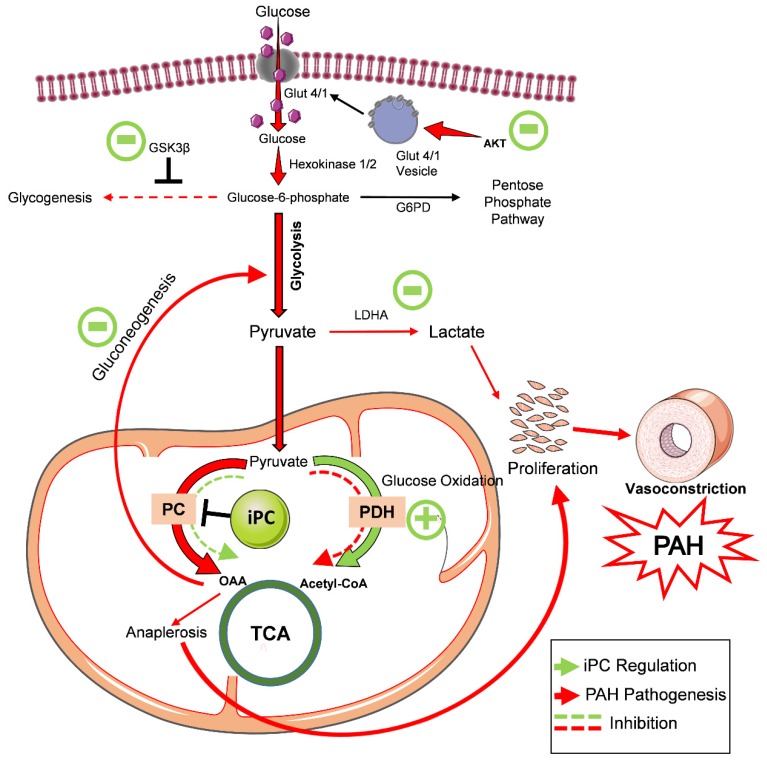
PC inhibitor attenuated glycolytic shift and metabolic reprogramming in pulmonary hypertension. In the sugen/hypoxic PAH model, phosphorylation of Akt upregulated cellular glucose uptake and glycolysis. This results in decreased oxidative phosphorylation, increased anaplerosis, and a reduction in glycogenesis. Increased anaplerosis and lactic acidosis feed-forward increased cellular proliferation and vascular remodeling in PAH. PC inhibitor treatment balanced cellular glucose influx, glycogen synthesis, and raised pentose phosphate pathway. As a result, glycolytic shift and improved oxidative phosphorylation returned back to control by inhibiting the key anaplerotic enzyme, PC, in PAH. Thus, proliferative pathological changes in PAH were found to be preserved with PC inhibitor treatment. PAH—pulmonary arterial hypertension, iPC–PC inhibitor, GSK3β–Glycogen synthase kinase 3 beta, G6PD–glucose-6 phosphate dehydrogenase, LDHA—lactate dehydrogenase—A, PC–pyruvate carboxylase, PDH—pyruvate dehydrogenase, OAA—oxaloacetate, TCA—tricarboxylic acid cycle, PAH—pulmonary arterial hypertension.

In the five-week sugen/hypoxic PAH model, we identify that the activation of Akt upregulated glycolysis. Both, Glut 4 and Glut 1 were translocated to the membrane, and this augmented cellular glucose influx. This results in increased aerobic glycolysis and anaplerosis, and a reduction in glycogenesis, similar to highly proliferative tumor cells [4]. Histological studies identified cellular proliferation and vascular remodeling in the lung tissue of PAH. These pathological modifications resulted in increased RV systolic pressure and Fulton index. Inhibition of anaplerosis by PC inhibitor, PAA, reduced the cellular glucose influx. This helped to balance glycolysis and oxidative phosphorylation back to the normal ratio. These observations suggest that PC is a mechanistic target in preventing PAH, and by inhibiting PC, it could be possible to attenuate proliferative pathological changes and pulmonary vascular remodeling in PAH (Figure 7).

Pulmonary vascular remodeling is the main cause of higher pulmonary vascular resistance and pulmonary arterial pressure. RV hypertrophy caused pressure overload is primarily considered as compensatory but eventually leads to RV dysfunction [14]. In association with the increased pressure, the lung tissue showed perivascular fibrosis and vasoconstriction in pulmonary arteries. Ki-67, a sensitive protein marker strongly associated with cellular proliferation [23], was found highly expressed in the PA wall. This cellular proliferation and fibrotic changes in the vascular wall clearly explains the cause of increased RV pressure in our disease model. In highly proliferative cells, due to their increased energy demand, PC functions to replenish anabolic intermediates to the TCA cycle [4]. Interestingly, PC inhibition demonstrated attenuation of vascular proliferation and RV pressure in the sugen/hypoxic model. These observations suggest that PC inhibition is an important target in preventing vascular proliferation and PAH pathogenesis.

Akt, also known as protein kinase B, plays a major role in hypoxia-induced pulmonary vascular resistance and remodeling [16]. Activation of Akt by phosphorylation at Serine-473 plays a major pathogenic contributor to many disease conditions, including PAH and human cancers [24]. We confirmed the Akt phosphorylation increased in the animal model. Akt activity promotes glucose metabolism by activating the translocation of Glut1 and Glut4 to the cell surface [25,26]. It was reported that the Warburg shift is a dominant vascular phenomenon in PAH and RV dysfunction [27]. Akt activation by phosphorylation can enhance cellular glucose uptake through the glucose transporters, Glut4 and Glut1, and can increase glycolysis in PAH [28]. In the present study, we profiled Glut4 and Glut 1 expression in different cellular fractions and found that it was increased in the membrane. As a result, glucose influx into cells was increased. High levels of intracellular glucose influx could upregulate the glycolytic metabolism to meet the demands of increased cellular proliferation. Proliferating cells tend to increase the expression of glucose transporters and glycolytic enzymes to compensate for increased metabolic needs [29]. Surprisingly, inhibition of PC and anaplerosis showed a significant effect on Akt phosphorylation. These observations suggest that the inhibition of PC, downstream of Akt mediated glucose metabolism [30], can possibly attenuate Akt activation by feedback regulation. The relation of Akt and PC should be explored in detail.

In the present study, the PAH disease model demonstrated increased glucose metabolism by stimulating the expression of hexokinase 1 and 2. HK1 is the first rate-limiting enzyme of the glycolytic pathway, and its expression can cause the downstream cascade amplification reaction to offer more ATP to proliferating cells [31]. Highly proliferative cancer-like cells demonstrated increased glycolytic metabolism through the increased expression of hexokinase 2, and also, Akt activation plays a critical role in the activation of glycolysis through hexokinase activation in cellular proliferation [17,32]. The final step of glycolysis, catalyzed by the enzyme pyruvate kinase (PKM), is a rate-limiting enzyme in glycolysis. Previous studies reported that highly dividing proliferative cancer cells show an increased PKM2/1 ratio in association with the Warburg effect [17]. Our sugen/hypoxic animals demonstrated an increased PKM2/1 ratio, representing the altered glycolytic shift and cellular proliferation. It was reported that the inhibition of anaplerotic pathway by blocking glutaminase, controlled glycolytic activity and decreased cellular proliferation [22]. Similarly, in our investigation, PC inhibition decreased hexokinase and pyruvate kinase expressions and thereby attenuated glycolytic shift and vascular cell proliferation in vivo.

GSK3β is a serine-threonine kinase regulating several cellular activities, like cell division, proliferation, and differentiation [33]. The expression of GSK3β was found to increase in disease. Elevation of GSK3β inversely correlates with glycogen synthase activity and glycogenesis [34]. Another parallel branch of glucose metabolism is the pentose phosphate pathway, which is mediated through the rate-limiting enzyme G6PD [35]. In the disease model, G6PD expression was found to increase in the lung tissue. Increased G6PD expression was found to be as a response to the glycolytic shift in pulmonary vascular cells related to cellular proliferation [36]. PC inhibition on the other hand, upregulated pentose phosphate pathway and glycogenesis. This could possibly help to counterbalance the increased glycolytic flux and return it to normal. In rapid glycolytic flux, the glycolytic enzyme LDHA converts pyruvate to lactate and stimulate glycolysis even further by oxidizing nicotinamide adenine dinucleotide (NADH) [37]. Increased LDHA expression may result in lactic acidosis, a well-reported event in PAH [19]. Interestingly, the inhibition of anaplerosis attenuated LDHA and directed pyruvate to mitochondrial oxidation.

Pyruvate metabolism is the central regulator of proliferative pathways, mediated through the inhibition of oxidative phosphorylation by decreasing PDH or the activation of anaplerosis by increasing PC. PDH is often considered as the mitochondrial sentinel enzyme, promoting pyruvate entry into oxidative phosphorylation [38,39]. Previous studies have reported that decreased PDH activity and Warburg effects can directly induce molecular and metabolic abnormalities in PAH [20]. Furthermore, in the PAH model, PDH expression was not significantly altered, but its activity showed a significant decrease, representing a reduction in acetyl-CoA and oxidative phosphorylation. Importantly, inhibition of PC with PAA increases the activity of the PDH, signifying an upregulation in glucose oxidation. These observations emphasize that the inhibition of PC in PAH could protect from the metabolic reprogramming from glycolysis to oxidative phosphorylation and prevent cellular proliferative changes. Interestingly, decreased PDH activity inversely acted with PC activation leading to increased OAA. OAA is a short-lived metabolite. However, our human PAH plasma samples showed increased stable oxalate, a degradative product of OAA, compared to other disease conditions such as diabetes or left heart diseases. Corroborating both the animal and the human data, we could perhaps enlist the idea that we can further use serum oxalate concentration as one of the biomarkers for PAH. Increased glycolysis and anaplerosis provides supplementary biomass for cellular proliferation in PAH. Thus, the inhibition of anaplerosis balanced the metabolic alterations back to normal.

The limitation of our study is based on focused research on the metabolic alterations of pulmonary vasculature and contribution of anaplerosis to vascular remodeling. It is possible that attenuation of anaplerosis via PC inhibition could lead to maladaptive response or remodeling of the right heart. The effect of PC inhibition on the right heart function should be explored in detail if PC inhibition would be considered for clinical use.

## 5. Conclusions

In conclusion, our findings suggest pyruvate carboxylase as a novel target to attenuate the glycolytic shift and increase glucose oxidation in PAH. PC inhibition attenuated Akt signaling, intracellular glycolysis, lactic acidosis, and anaplerosis. Suppressing cellular proliferation by inhibiting anaplerosis prevents further development of pulmonary vascular remodeling. Thus, the findings obtained from our study suggest that the inhibition of anaplerosis is an effective therapeutic target in the normalization of the metabolic reprogramming in PAH.

## Supplementary Materials

The following are available online at https://www.mdpi.com/2077-0383/9/2/443/s1, Figure S1: PC inhibitor attenuated sugen/hypoxia induced inflammation.
