# Supplementary material for: Inhibition of Anaplerosis Attenuated Vascular Proliferation in Pulmonary Arterial Hypertension
Source: J Clin Med. 2020 Feb 6;9(2):443. doi: 10.3390/jcm9020443 (PMC7074087; doi:10.3390/jcm9020443)
Supplement: Supplementary file 1 [file jcm-09-00443-s001.pdf]

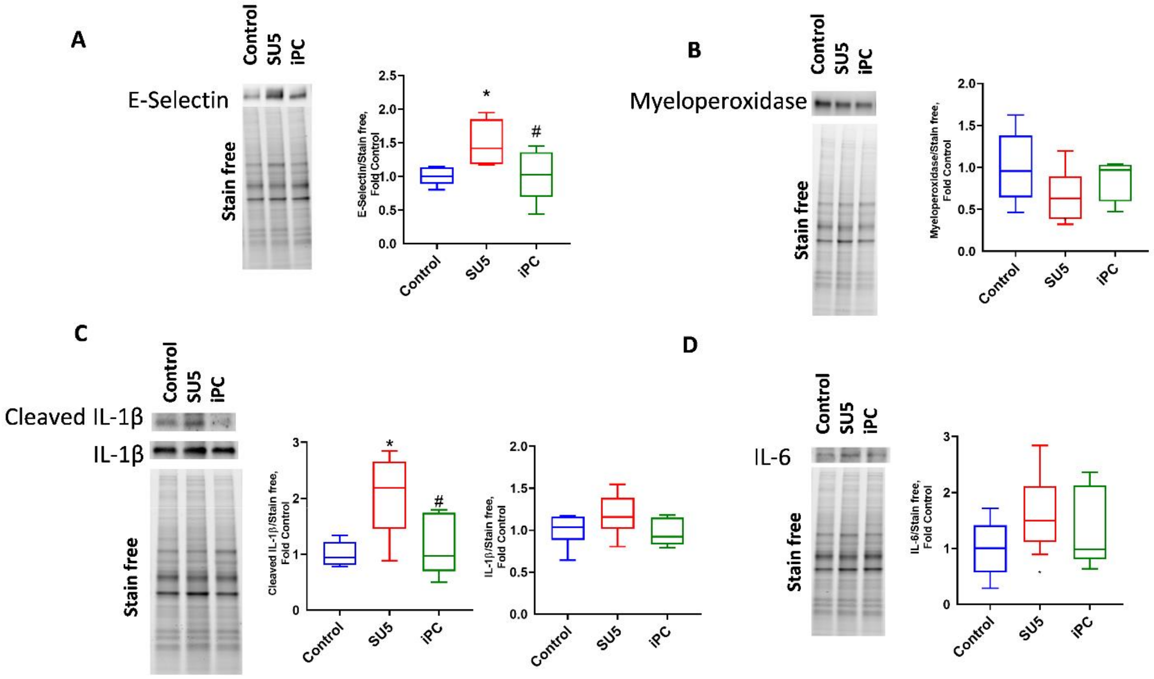

Supplementary Figure 1. PC inhibitor attenuated sugen/hypoxia induced inflammation. (**A**) E-selectin expression upregulated in PAH model was found reduced with PC inhibitor treatment. (**B**) Peroxidase enzyme myeloperoxidase was not significantly altered with either PAH induction or PC inhibitor treatment. (**C**) Inflammatory mediator interleukin (IL)1β (cleaved) was found significantly increased in sugen/hypoxic lung tissue and attenuated with PC inhibitor. However, full length transcript (pro-IL1β) did not show any significant changes. (**D**) Interleukin-6 was not significantly altered in lung tissue of PAH and PAA treatment group. Data expressed as box-and-whisker plot with median value ±MinMax, N=5-6, * $p < 0.05$ versus control, # $p < 0.05$ versus SU5 by one way ANOVA. Antibody and dilution details: IL-1 β (1:1000) from Abcam (Ab9722); IL-6 (1:500) from Sata Cruz (sc-57315); E-selectin (1:300) from ProteinTech (20894-1-AP); Anti-Myeloperoxidase (1:1000) from Abcam (ab208670).
